# SCOWLP update: 3D classification of protein-protein, -peptide, -saccharide and -nucleic acid interactions, and structure-based binding inferences across folds

**DOI:** 10.1186/1471-2105-12-398

**Published:** 2011-10-13

**Authors:** Joan Teyra, Sergey A Samsonov, Sven Schreiber, M Teresa Pisabarro

**Affiliations:** 1 Structural Bioinformatics BIOTEC TU Dresden, Tatzberg 47-51 01037 Dresden, Germany; 2 Applied Bioinformatics Technology Platform, BIOTEC TU Dresden, Tatzberg 47-51 01037 Dresden, Germany

## Abstract

**Background:**

Protein interactions are essential for coordinating cellular functions. Proteomic studies have already elucidated a huge amount of protein-protein interactions that require detailed functional analysis. Understanding the structural basis of each individual interaction through their structural determination is necessary, yet an unfeasible task. Therefore, computational tools able to predict protein binding regions and recognition modes are required to rationalize putative molecular functions for proteins. With this aim, we previously created SCOWLP, a structural classification of protein binding regions at protein family level, based on the information obtained from high-resolution 3D protein-protein and protein-peptide complexes.

**Description:**

We present here a new version of SCOWLP that has been enhanced by the inclusion of protein-nucleic acid and protein-saccharide interactions. SCOWLP takes interfacial solvent into account for a detailed characterization of protein interactions. In addition, the binding regions obtained per protein family have been enriched by the inclusion of predicted binding regions, which have been inferred from structurally related proteins across all existing folds. These inferences might become very useful to suggest novel recognition regions and compare structurally similar interfaces from different families.

**Conclusions:**

The updated SCOWLP has new functionalities that allow both, detection and comparison of protein regions recognizing different types of ligands, which include other proteins, peptides, nucleic acids and saccharides, within a solvated environment. Currently, SCOWLP allows the analysis of predicted protein binding regions based on structure-based inferences across fold space. These predictions may have a unique potential in assisting protein docking, in providing insights into protein interaction networks, and in guiding rational engineering of protein ligands. The newly designed SCOWLP web application has an improved user-friendly interface that facilitates its usage, and is available at http://www.scowlp.org.

## Background

Proteins are ubiquitous and interact with other molecules to perform their function, being conditioned to timing and location [1]. High-throughput technologies for the identification of protein interactions are generating a plethora of new data that should be independently studied to decipher the specific molecular role of the proteins and their cellular functions [2]. Structural determination methods at atomic resolution are indispensable for the functional characterization of protein interactions. However, techniques for isolating protein complexes and their structural determination are still encountering many challenges, and hence, experimental structural studies are not always possible. Alternatively, the rapid accumulation of protein complex structures in the PDB repository [3,4] provides an unprecedented opportunity for comparative analysis of protein interactions that can be used to predict binding regions and modes [5], to model protein complexes [6], and to improve our understanding of the principles governing protein recognition [7]. To facilitate these comparative studies, it is necessary to generate tools that can allow us to analyze the available experimental structures of protein complexes [8]. In fact, several databases have been developed to structurally identify and classify protein-protein and protein-peptide interactions at family level, such as 3DID [9], SCOPPI [10] and SCOWLP [11]. Their classifications are based on collecting all interacting information per protein family, then calculating the binding residues similarity, and finally clustering the different binding regions and their partners. Another database, IBIS [12], contains binding regions and interacting partners inferred from the inspection of complexes formed by close homologous proteins instead of using structural classification schemes. Unlike the others, SCOWLP has been developed towards an atomic inspection of the interactions by applying physicochemical principles and by considering water molecules in protein interfaces, since solvent has been shown to be abundant and important in the mediation of protein interactions [13,14], and to improve protein contact predictions [15] and docking [16].

SCOWLP is a database and a web application containing a structural classification of protein binding regions at SCOP family level, including protein-protein and peptide interactions [11]. In the new updated version we present here, we additionally include two biologically relevant protein ligands that are quite abundant in the PDB: saccharides (SAC) and nucleic acids (NA) [17-20]. We also considered solvent in the definition of protein interactions, since it has been shown to be critical mediating both, protein-NA [21] and protein-SAC [22] interactions, highlighting the importance of the new SCOWLP to perform in-detail inspection of these kind of interactions. Another novelty in SCOWLP is the inclusion of predicted binding regions for each protein family. These predictions are inferred from significantly conserved binding regions belonging to structurally similar protein families independently of their fold [5,23]. It has been observed that proteins with different folds and functions can recognize molecules through binding regions containing similar local structural features or interacting motifs [24-26]. Therefore, the predicted binding inferences might become very useful to suggest alternative recognition regions for a protein family and to compare structurally similar binding regions from different families.

In summary, the updated SCOWLP classification with its newly designed web application represent a unique framework for the identification and comparative analysis of protein binding regions at atomic level. In the following sections, we explain the methodology used to build the database, and describe the architecture and possible usages of the web application.

## Construction and Content

The new version of SCOWLP contains protein interactions with different ligand types, including proteins, peptides, nucleic acids (NA) and saccharides (SAC), taking into account interfacial solvent mediating protein interactions. Interacting residues and molecules are described at physicochemical level according to atom type and distance criteria. The following types of interactions are considered: hydrogen bonds, with distance donor/acceptor atom ≤ 3.6 Å; salt bridges, with charged atom distance ≤ 4 Å; Van der Waals, with atoms at distance ≤ 4.5 Å. Water-mediated residue interactions through a water molecule are also considered in the interface definition. The specific definition of the ligand types, and the protein interfaces is as follows:

• Protein-protein interactions: The 4,194 protein families from SCOP V1.75 [27] are used to define protein domain boundaries within PDB files.

• Protein-peptide interactions: All PDB chains that are labeled "ATOM", not defined in SCOP, and shorter than 90 residues are considered peptides [28].

• Protein-nucleic acids interactions: PDB residues labeled as standard nucleotides are selected. We differentiate RNA form DNA by the presence of the O2' group in the ribose ring. Nucleic acid chains are merged in a single unit (double strand) if there is at least one inter-base atomic interaction among chains.

• Protein-saccharide interactions: The SAC molecules are extracted from PDB files labeled with the terms "saccharide", "carbohydrate" and/or "sugar", and containing HETATM atoms. We obtained 307 unique molecules (three-letter code) that include neither standard or modified nucleotides, nor SAC modifications bigger than the SAC moieties. The oligosaccharide units can be represented in the PDB either within a common HETATM type or as a collection of them. In the later case, SAC units are identified and merged together in a single oligosaccharide molecule using the PDB connectivity. SAC connectivity to protein domains is also checked to differentiate covalent (intra) and non-covalent (inter) protein interactions.

SCOWLP currently contains 97,252 protein-protein, 3,563 protein-peptide, 2,568 protein-NA (1,660 DNA, 908 RNA) and 10,590 protein-SAC complexes. Crystal packing contacts are filtered out using a support vector machine-based program, NOXclass [29] (cutoff 70%), which takes into account the distinctive properties of these protein contacts [30].

The classification of family binding regions has been performed by clustering interacting domains based on binding region similarities. As described previously [11], this value has been obtained based on the interacting residues overlap once they are mapped onto the structure-based sequence alignments of the family members. Likewise, the inferred binding regions are obtained among members of different families aligned using non-sequential structural alignments as previously described [5]. SCOWLP contains a total of 7,121 protein binding regions identified at zero similarity cutoff; from which 2,315 have more than one interface. In addition, it contains 8,985 predicted binding regions, 786 of them in protein families that so far lack binding information in the PDB.

## Utility & Discussion

### Web architecture

SCOWLP web application follows the SCOP hierarchical levels to classify protein structures: RT-root, CF - class family, SF - super family, where families are finally listed. In addition, it extends the SCOP classification with three protein interaction levels: FA - family, BR - binding region and IF - interface. FA level contains a list of binding regions, defined as distinctive surface regions of a protein family used to recognize other molecules. BR level contains a list of interfaces distinguishing the different partners or ligands that a specific region can recognize. IF level contains a list of domains interacting with the same ligand, and that are linked to their original PDB file (e.g. 2oei:AB). Each binding region and interface is represented by identifiers (BR_24483 or IF_24486), since their association to an automatic description is not possible.

### Query

SCOWLP web application facilitates the hierarchical navigation through the different levels. It also contains a keyword search box for SCOP descriptions, PDB Ids and similar SCOP domain sequences identified using the BLAST algorithm. Some specific examples of the query capabilities are shown in the SCOWLP main page.

### Search options

**Figure 1:**
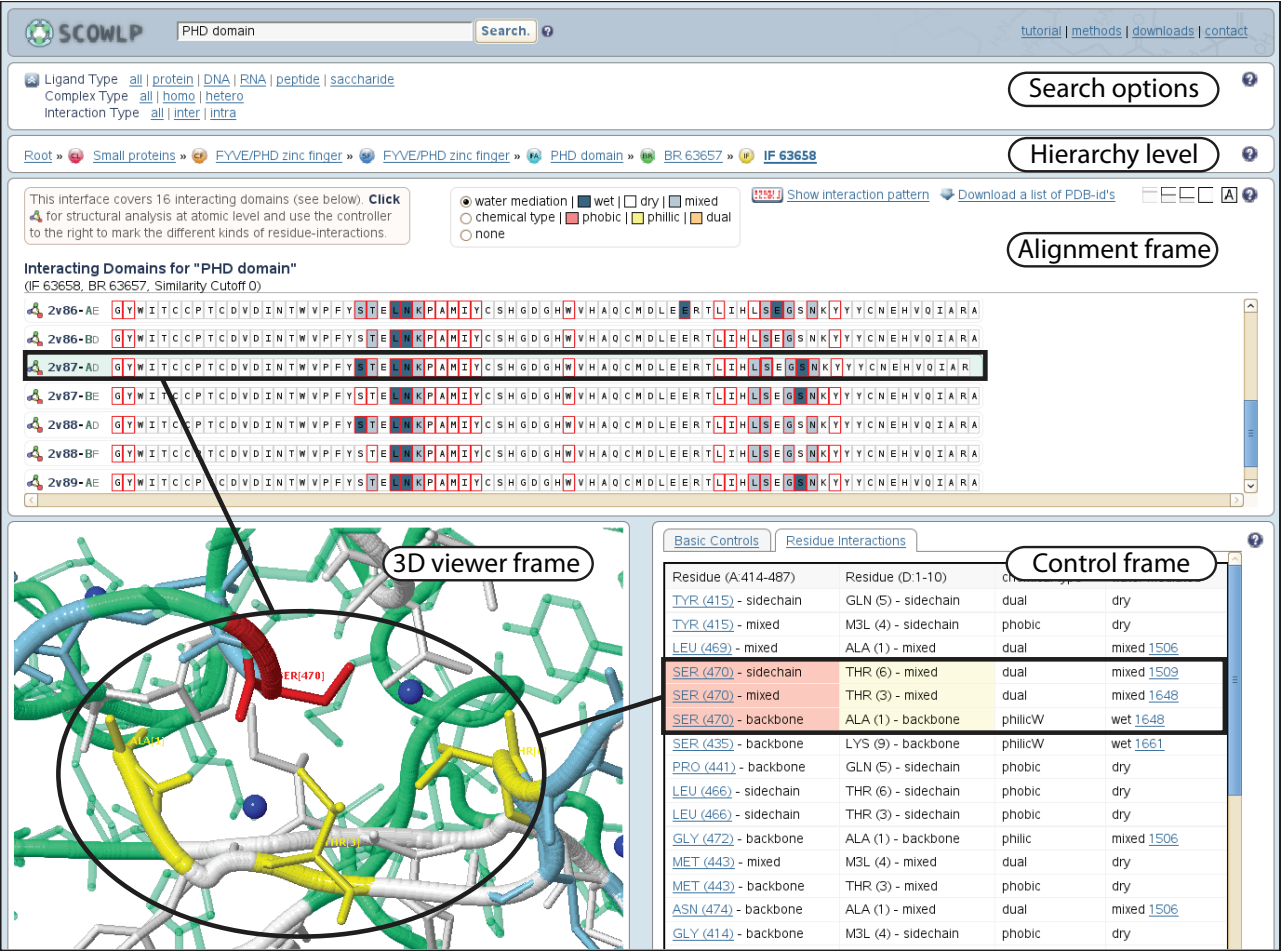
**SCOWLP architecture**; Web application snapshot at the Interface level for PHD domain interacting with a peptide ligand. The search options for filtering the information, and the hierarchy level to facilitate the navigation are shown. In addition, the three interconnected frames for data analysis are also highlighted: Alignment, 3D Viewer and Control frames. A residue selected either in alignment or control frames (black boxes) is automatically highlighted in the 3D Viewer (circle).

The interacting levels of SCOWLP (FA, BR, IF) contain the Search options located at the top of the web application (Figure 1). This feature reduces the query and navigation to a specific list of interacting domains based on: i) **Ligand type: **proteins, peptides, DNA, RNA and saccharides; ii) **Complex type: **same (homo) or different (hetero) domain families; iii) **Interaction type: **same (intra) or to different (inter) domain chains; iv) **Clustering cutoff: **four cutoff values that define the final binding region clusters per family, as described in ref. [5] (default cutoff is zero). Note that the possibility of filtering out homo-dimers and intra-domain interactions may become very useful since their number is rather high and irrelevant for many analyses.

### Data analysis

The information at each interacting level is displayed in the web page in consecutive steps. Each level shares a common web page composed by three interconnected frames to facilitate the analysis of the information (Figure 1):

**• Alignment frame: **The structure-based sequence alignments of the corresponding domains are shown in each interacting level. In addition, the FA level also includes predicted binding regions, whereas BR level includes predicted interfaces, information inferred from other structurally-related protein families. The interacting residues are highlighted for better analysis of binding patterns. At IF level, these residues can be colored by their physicochemical properties (hydrophobic or hydrophilic), and by the water participation in the interactions (dry, wet or dual). The patterns and the physicochemical properties facilitate the distinction between conserved and variable interactions. The structure of each member can be visualized in the 3D Viewer frame upon click selection of the Jmol icon.

**• 3D Viewer frame: **The Jmol plug-in [31] is available for 3D visualization of the members shown in the Alignment frame as follows: The FA level displays a surface representation of the binding regions onto a representative structure for general visualization of the spatial locations used for recognition. The BR level allows the 3D visualization of the different interfaces containing different ligands and/or binding modes. The IF level, allows the user to visualize atomic details of all domains interacting with a common interface and to label them according to the physicochemical and solvent criteria selected in the alignment frame. Subtle structural differences within domains interacting with the same interface can be detected and analyzed.

**• Control frame: **This frame contains Jmol-interactive commands and includes links to the PDB and FA levels. In addition, the residue-residue interaction list is displayed with their physicochemical and water-mediation properties for each interacting domains.

The **Frame Interconnectivity **feature implemented in the IF level of the new SCOWLP allows the possibility to automatically highlight (i.e. centered zoom and color) a specific interacting residue in the 3D structure of the viewer upon clicking either the Alignment or the Control frame (Figure 1).

### Applications

The main page contains examples of the SCOWLP main functionalities: i) exploration of the different surface regions that a protein family uses to recognize other molecules, ii) identification of the different ligands that a given region can recognize, and iii) comparative analysis of the interacting properties of a group of domains in complex with the same ligand. These analyses include the conservation and variability of not only interfacial residues but of their interactions, taking into consideration water-mediated interactions.

**Figure 2:**
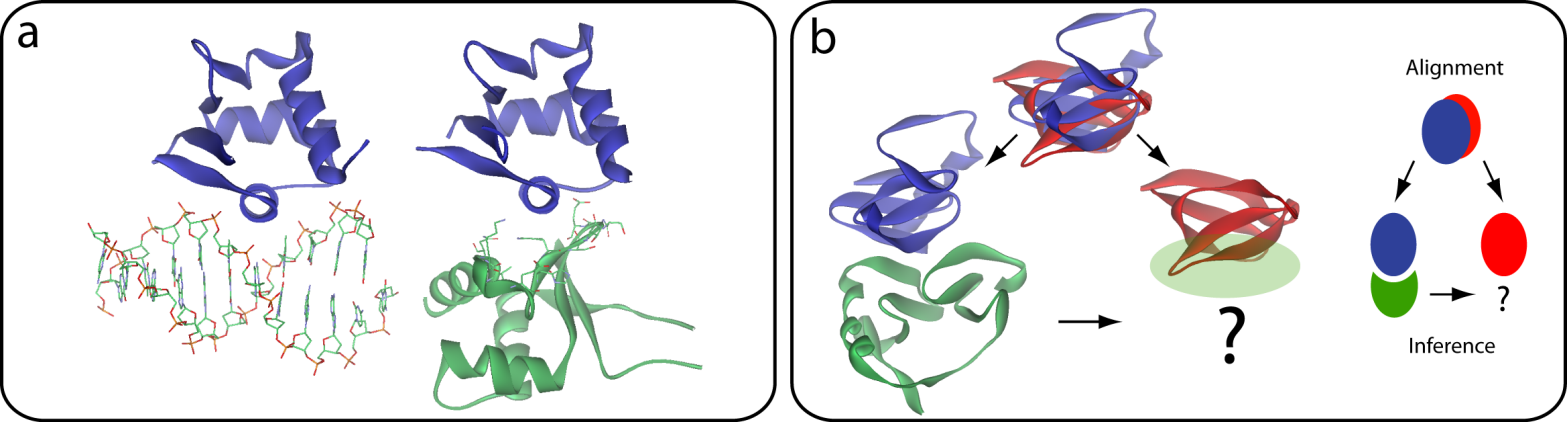
**SCOWLP applications**: a) The same binding region of the protein family "heat-shock transcription factor" (blue ribbon) presents interfaces for DNA (left, pdb:3hts) and protein (right, pdb:1fbq); b) Schematic representation of the method used to infer binding regions and interfaces. Two proteins from different SCOP families (violet and red) present structural resemblances. Since violet protein interacts with its green ligand through a region that is conserved in the violet-red structural alignment, violet's binding region is inferred to the red protein, suggesting that it could also recognize other proteins through this inferred region in a similar binding mode to violet-green interface.

It is important to highlight that one of the main potentials of the new SCOWLP relies on the rapid identification of protein families able to recognize one or several ligand types through the same region. For instance, the selection of a specific combination of ligands in the search options ("protein and DNA") will show only those SCOP families that can recognize proteins and DNA thorough the same surface region. An example is the "heat-shock transcription factor" (search by keyword), that has a binding region at FA level (BR_1892) recognizing these two ligands. By clicking at the BR Id, two different interfaces are shown for this family binding region, one recognizing a protein and the other DNA, and therefore, responsible of different functions (Figure 2a). After clicking at the IF Id, the web application also allows the analysis of the interacting features governing the two interfaces.

Another key feature of the new SCOWLP is the possibility to obtain alternative binding regions and interfaces, as schematically shown in Figure 2b. These predicted binding regions are inferred from other structurally similar protein families. For instance, if we select in the Search options for "Binding region type: only predicted" we will filter those SCOP families that do not have any binding information available in the PDB yet (i.e. no structures in complex with other molecules), and that present predicted binding regions (786 families). Navigating through the SCOP hierarchy, the predicted binding regions for any of these families can be explored. An example is the "DEATH effector domain, DED" (search by keyword), where three different binding regions have been inferred. Detailed analysis of the binding regions (3D Viewer) and the structurally similar proteins and their ligands (Control frame) might be useful to explore putative binding regions and ligands for DED domain.

The number of protein-protein interactions obtained from large scale technologies is increasing [32], though protein-protein interaction networks contain a considerable amount of noise due to intrinsic errors [2]. Structural information has already been implemented into these networks in order to distinguish direct vs. indirect interactions between proteins, and competing vs. complementary interactions, whether two proteins interact to a third one through the same or a different binding region [8,33]. The structural classification and the predicted protein binding regions contained in SCOWLP might contribute towards a more accurate construction of protein-protein interaction networks.

In summary, the examples explained above point out the unique potential of SCOWLP for identification, analysis and prediction of protein interactions. Our ultimate goal is to facilitate the analysis of protein interactions that may contribute to a better understanding of the rules governing protein recognition and molecular function.

## Conclusions

Here we present an updated and enhanced version of the SCOWLP database and its user-friendly web application. The new SCOWLP comprises its previous structural classification of all protein binding regions of the PDB at protein family level, including protein-peptide and water-mediated interactions, which has been enhanced by the inclusion of protein-nucleic acid and protein-saccharide interactions. In addition, the original functionality of SCOWLP towards the prediction of protein binding regions has been augmented by the inclusion of binding regions inferred from structurally similar proteins across fold space. The new SCOWLP database and its newly designed web application, which includes new helpful features such as frame interconnectivity, represent useful tools for the detailed analysis of the protein interactome. They provide the user a valuable assistance in suggesting protein recognition regions and comparing structurally similar interfaces from different protein families, which denotes their unique potential for gaining a better understanding of protein interaction networks and for guiding protein docking and rational ligand design.

## Availability & Requirements

SCOWLP database and web application are freely available at http://www.scowlp.org. MySQL files containing the entire database can be downloaded for independent studies. SCOWLP classification is updated with each new SCOP release. Programming language: Oracle Java 6.0, Javascript. Requirements-serverside: Oracle Java 6.0 (or higher), Apache Tomcat 6.0 (or higher). Requirements-clientside: Oracle Java 6.0 (or higher), Mozilla Firefox 3.6 (or higher), Google Chrome 13 (or higher). Software source code will be provided upon request for non commercial usage.

## Competing interests

The authors declare that they have no competing interests.

## Authors' contributions

JT carried out the study. SAS participated in the identification of saccharides and their interactions. SS and JT developed the new web application. MTP coordinated the study. MTP and JT wrote the manuscript. All authors read and approved the final manuscript.

## References

[B1] Yamada T Bork P Evolution of biomolecular networks: lessons from metabolic and protein interactions Nature Reviews Molecular Cell Biology 2009 10 11 791 803 10.1038/nrm2787 19851337

[B2] Gentleman R Huber W Making the most of high-throughput protein-interaction data Genome Biology 2007 8 10 112 10.1186/gb-2007-8-10-112 18001486 PMC2246275

[B3] PDB http://www.rcsb.org/pdb

[B4] Aloy P Russell RB Ten thousand interactions for the molecular biologist Nature Biotechnology 2004 22 10 1317 1321 10.1038/nbt1018 15470473

[B5] Teyra J Hawkins J Zhu H Pisabarro MT Studies on the inference of protein binding regions across fold space based on structural similarities Proteins 2011 79 2 499 508 10.1002/prot.22897 21069715

[B6] Kiel C Beltrao P Serrano L Analyzing protein interaction networks using structural information Annual Review of Biochemistry 2008 77 415 441 10.1146/annurev.biochem.77.062706.133317 18304007

[B7] Jones S Thornton JM Principles of protein-protein interactions Proc Natl Acad Sci USA 1996 93 1 13 20 10.1073/pnas.93.1.13 8552589 PMC40170

[B8] Aloy P Russell RB Structural systems biology: modelling protein interactions Nature Reviews Molecular Cell Biology 2006 7 3 188 197 10.1038/nrm1859 16496021

[B9] Stein A Ceol A Aloy P 3did: identification and classification of domain-based interactions of known three-dimensional structure Nucleic Acids Res 2011 39 Database D718 723 Epub 2010 Oct 2021 10.1093/nar/gkq962 PMC3013799 20965963

[B10] Winter C Henschel A Kim WK Schroeder M SCOPPI: a structural classification of protein-protein interfaces Nucleic Acids Res 2006 34 Database D310 314 10.1093/nar/gkj099 PMC1347461 16381874

[B11] Teyra J Paszkowski-Rogacz M Anders G Pisabarro MT SCOWLP classification: structural comparison and analysis of protein binding regions BMC Bioinformatics 2008 9 9 10.1186/1471-2105-9-9 18182098 PMC2259299

[B12] Shoemaker BA Zhang D Thangudu RR Tyagi M Fong JH Marchler-Bauer A Bryant SH Madej T Panchenko AR Inferred Biomolecular Interaction Server--a web server to analyze and predict protein interacting partners and binding sites Nucleic Acids Res 2009 38 Database D518 524 Epub 2009 Oct 2020 10.1093/nar/gkp842 PMC2808861 19843613

[B13] Teyra J Pisabarro MT Characterization of interfacial solvent in protein complexes and contribution of wet spots to the interface description Proteins 2007 67 4 1087 1095 10.1002/prot.21394 17397062

[B14] Samsonov S Teyra J Pisabarro MT A molecular dynamics approach to study the importance of solvent in protein interactions Proteins 2008 73 2 515 525 10.1002/prot.22076 18452208

[B15] Samsonov SA Teyra J Anders G Pisabarro MT Analysis of the impact of solvent on contacts prediction in proteins BMC Structural Biology 2009 9 22 10.1186/1472-6807-9-22 19368710 PMC2676287

[B16] van Dijk AD Bonvin AM Solvated docking: introducing water into the modelling of biomolecular complexes Bioinformatics 2006 22 19 2340 2347 10.1093/bioinformatics/btl395 16899489

[B17] Timmer MS Stocker BL Seeberger PH Probing glycomics Current Opinion in Chemical Biology 2007 11 1 59 65 10.1016/j.cbpa.2006.11.040 17208037

[B18] Kim TH Ren B Genome-wide analysis of protein-DNA interactions Annual Review of Genomics & Human Genetics 2006 7 81 102 10.1146/annurev.genom.7.080505.115634 16722805

[B19] Lee S Blundell TL BIPA: a database for protein-nucleic acid interaction in 3D structures Bioinformatics 2009 25 12 1559 1560 10.1093/bioinformatics/btp243 19357098

[B20] Ranzinger R Herget S Wetter T von der Lieth CW GlycomeDB - integration of open-access carbohydrate structure databases BMC Bioinformatics 2008 9 384 10.1186/1471-2105-9-384 18803830 PMC2567997

[B21] Jayaram B Jain T The role of water in protein-DNA recognition Annual Review of Biophysics & Biomolecular Structure 2004 33 343 361 10.1146/annurev.biophys.33.110502.140414 15139817

[B22] Tschampel SM Woods RJ Quantifying the role of water in protein-carbohydrate interactions J Phys Chem A 2003 107 43 9175 9181 10.1021/jp035027u 16906231 PMC1538976

[B23] Slabicki M Theis M Krastev DB Samsonov S Mundwiller E Junqueira M Paszkowski-Rogacz M Teyra J Heninger AK Poser I A genome-scale DNA repair RNAi screen identifies SPG48 as a novel gene associated with hereditary spastic paraplegia PLoS Biology 2010 8 6 e1000408 10.1371/journal.pbio.1000408 20613862 PMC2893954

[B24] Keskin O Nussinov R Favorable scaffolds: proteins with different sequence, structure and function may associate in similar ways Protein Eng Des Sel 2005 18 1 11 24 10.1093/protein/gzh095 15790576

[B25] Gao M Skolnick J Structural space of protein-protein interfaces is degenerate, close to complete, and highly connected Proc Natl Acad Sci USA 2010 107 52 22517 22522 Epub 22010 Dec 22513 10.1073/pnas.1012820107 21149688 PMC3012513

[B26] Ogmen U Keskin O Aytuna AS Nussinov R Gursoy A PRISM: protein interactions by structural matching Nucleic Acids Res 2005 33 Web Server W331 336 10.1093/nar/gki585 PMC1160261 15991339

[B27] Lo Conte L Ailey B Hubbard TJ Brenner SE Murzin AG Chothia C SCOP: a structural classification of proteins database Nucleic Acids Res 2000 28 1 257 259 10.1093/nar/28.1.257 10592240 PMC102479

[B28] Teyra J Doms A Schroeder M Pisabarro MT SCOWLP: a web-based database for detailed characterization and visualization of protein interfaces BMC Bioinformatics 2006 7 1 104 10.1186/1471-2105-7-104 16512892 PMC1459204

[B29] Zhu H Domingues FS Sommer I Lengauer T NOXclass: prediction of protein-protein interaction types BMC Bioinformatics 2006 7 27 10.1186/1471-2105-7-27 16423290 PMC1386716

[B30] Carugo O Argos P Protein-protein crystal-packing contacts Protein Science 1997 6 10 2261 2263 9336849 10.1002/pro.5560061021 PMC2143556

[B31] Jmol http://jmol.sourceforge.net

[B32] Charbonnier S Gallego O Gavin AC The social network of a cell: recent advances in interactome mapping Biotechnol Annu Rev 2008 14 1 28 18606358 10.1016/S1387-2656(08)00001-X

[B33] Kim PM Lu LJ Xia Y Gerstein MB Relating three-dimensional structures to protein networks provides evolutionary insights Science 2006 314 5807 1938 1941 10.1126/science.1136174 17185604

